# Carbonic Anhydrase Inhibitors. Part 91. Metal Complexes of Heterocyclic Sulfonamides as Potential Pharmacological Agents in the Treatment of Gastric Acid Secretion Imbalances

**DOI:** 10.1155/MBD.2000.57

**Published:** 2000

**Authors:** Marc A. Ilies, Claudiu T. Supuran, Andrea Scozzafava

**Affiliations:** 1 University of Agricultural Sciences and Veterinary Medicine Faculty of Biotechnologies Department of Chemistry B-dul Marasti hr. 59 Bucharest 71331 Romania; 2 Università degli Studi Dipartamento di Chimica Laboratorio di Chimica Inorganica e Bioinorganica Via Gino Capponi 7 Firenze I-50121 Italy

## Abstract

Zinc, magnesium, aluminum and copper complexes of several potent, clinically used carbonic anhydrase (CA) sulfonamide inhibitors, such as acetazolamide, methazolamide, ethoxzolamide and benzolamide were tested for their possible applications as antacids, in experimental animals. Gastric acid secretion parameters 3 days after treatment with these CA inhibitors (2 × 500 mg, twice a day), in dogs with chronic gastric fistulas, led to the observation that the gastric acid parameters BAO (the basal acid output), and MAO (the maximal acid output after stimulation with histamine) were drastically reduced, as compared to the same parameters in animals that did not receive these enzyme inhibitors. These are promising results for the possible use of metal complexes of heterocyclic sulfonamides as treatment alternatives (alone or in combination with other drugs) for gastric acid secretion imbalances.


### CARBONIC ANHYDRASE INHIBITORS. PART 91. METAL COMPLEXES OF HETEROCYCLIC SULFONAMIDES AS POTENTIAL PHARMACOLOGICAL AGENTS IN THE TREATMENT OF GASTRIC ACID SECRETION IMBALANCES

Marc A. Ilies,[1] Claudiu T. Supuran*[2] and Andrea Scozzafava[2]

[1] University of Agricultural Sciences and Veterinary Medicine, Faculty of Biotechnologies, Department of Chemistry, B-dul Marasti nr. 59, 71331-Bucharest, Roumania

[2] Università degli Studi, Dipartamento di Chimica, Laboratorio di Chimica Inorganica e Bioinorganica, Via Gino Capponi 7, I-50121, Firenze, Italia; E-mail: cts@bio.polosci.unifi.it

**Abstract**: Zinc, magnesium, aluminum and copper complexes of several potent, clinically used carbonic anhydrase (CA) sulfonamide inhibitors, such as acetazolamide, methazolamide, ethoxzolamide and benzolamide were tested for their possible applications as antacids, in experimental animals. Gastric acid secretion parameters 3 days after treatment with these CA inhibitors (2 x 500 mg, twice a day), in dogs with chronic gastric fistulas, led to the observation that the gastric acid parameters BAO (the basal acid output), and MAO (the maximal acid output after stimulation with histamine) were drastically reduced, as compared to the same parameters in animals that did not receive these enzyme inhibitors. These are promising results for the possible use of metal complexes of heterocyclic sulfonamides as treatment alternatives (alone or in combination with other drugs) for gastric acid secretion imbalances.

### Introduction

Carbonic anhydrase (CA, EC 4.2.1.1) together with $H^+/K^+$-ATP-ase are the key enzymes involved in gastric acid secretion.[1-4] $H^+$ ions needed for gastric HCl formation are generated either by hydration of $CO_2$, a reaction catalyzed by at least two CA isozymes present in gastric parietal cells (CA II – cytosolic, and CA IV - membrane-bound form, reaction *1*), or by ATP hydrolysis – (reaction 2), in the presence of $H^+/K^+$-ATP-ase:[1-4]

$$CO_2 + H_2O \rightleftharpoons HCO_3^- + H^+ \qquad (1)$$

$$ATP^{4-} + H_2O \rightleftharpoons ADP^{3-} + HPO_4^{2-} + H^+ \qquad (2)$$

It has been proved that high doses of acetazolamide (5-acetamido-1,3,4-thiadiazole-2-sulfonamide **1**), a clinically used sulfonamide CA inhibitor,[6] constitute a useful therapy of gastro-duodenal ulcers,[1c,5] although side effects correlated with CA inhibition in other tissues than the stomach impeded its wide clinical applications. The major side effects of acetazolamide as antiulcer drug are correlated with the massive doses needed for efficient inhibition of gastric CA (around 4 g/day),[1c] and they include: mild paresthesias of the limbs, slight asthenia and somnolence, enhanced diuresis (in the first 3 days of treatment), weight loss.[1c] Mention should be made that the frequency and severity of such side effects are highly decreased in glaucoma patients taking acetazolamide (as systemic intraocular pressure lowering drug),[1d,e] since the doses needed for the ophthalmologic effect of the drug are much more reduced (0.5 – 1.0 g/day) as compared to the amount needed for anti-ulcer effects (4 g/day).[1c]

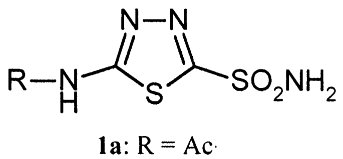

**1a**: R = Ac
**1b**: R = $PhSO_2$

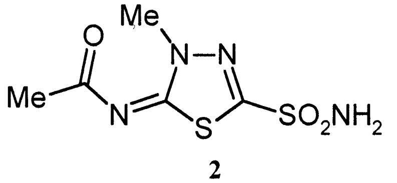

**2**

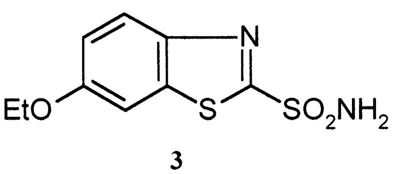

**3**

Metal complexes of heterocyclic sulfonamides such as acetazolamide **1a**, methazolamide **2**, ethoxzolamide **3** or benzolamide **1b** (all clinically used CA inhibitors)[6] reported previously by our group, have been shown to possess very efficient CA inhibitory properties,[7-9] and their mechanism of action has been explained as being due to both sulfonamidate anions as well as metal ions (formed after dissociation of the complex in dilute solutions) which interact thereafter with different binding sites of the enzyme.[10]

On the other hand, many pharmacological preparations of the antacid type contain in addition to sodium bicarbonate (as $H^+$ neutralizing agent) also Zn(II), Mg(II) and/or Al(III) compounds (mainly as oxides or hydroxides).[11] It appeared thus of interest to investigate whether some of the above-mentioned metal complexes (alone or in combination), containing the sulfonamides **1-3** as well as the these metal ions (as well as Cu(II), for reasons that will be explained shortly, would possess properties useful for their development as antacids, since in addition to the acid neutralizing effects due to their reaction with $H^+$ ions present in gastric juice, by their strong CA inhibitory properties they might interfere with gastric acid secretion by two mechanism of actions: (i) neutralization reactions of the type: $(RSO_2NH)_nM + n\ H^+ \rightarrow n\ RSO_2NH_2 + M^{n+}$ ;and (ii) CA inhibition by sulfonamides and/or sulfonamide complexes, with the reduction of $H^+$ ions generated accordingly to reaction (*1*). This hypothesis has been verified by some in vivo studies (in dogs with gastric fistula) reported here. The animals received some of the Zn(II), Mg(II), Al(III) and Cu(II) complexes of sulfonamides **1-3**, and their gastric acid secretion parameters were controlled prior and after the treatment. Parameters such as BAO (basal acid output), and MAO (maximal acid output after stimulation with histamine) were drastically reduced in the treated animals, as compared to the same parameters in animals that did not receive these enzyme inhibitors.

### Material and Methods

Sulfonamides used for the preparation of coordination compounds (acetazolamide **1a**, and methazolamide **2**) were from Sigma-Àldrich, benzolamide **1b** was prepared as described in ref.[12], whereas ethoxzolamide **3** as described in ref.[13] Metal salts and solvents were from Sigma-Aldrich and were used without further purification. Human isozymes hCA I and II, and bCA IV were prepared as described previously.[1e,d] The metal complexes used for the pharmacological evaluations were prepared as described in previous works of this group.[7-10] Buffers and 4-nitrophenyl acetate were from Sigma. Inhibitors were assayed spectrophotometrically at 400 nm, by the esterase method,[14] for the inhibition of 4-nitrophenyl acetate hydrolysis catalyzed by the three CA isozymes. The buffer used in the enzymatic assay (Tris-$H_2SO_4$ 10 mM, pH 7.8) was brought to an ionic strength $\mu = 0.1$, by addition of $K_2SO_4$. Enzyme concentrations used in the experiments were 1.7 nM for hCA II, and 24 nM for bCA IV. Stock solutions of inhibitors (1 mM) were obtained in DMSO, and dilutions up to 0.01 nM were done thereafter with distilled deionized water. Enzyme and inhibitor solutions were preincubated for 15 min prior to assay.

*Ex vivo* CA activity was determined in gastric mucosa homogenates as follows: precisely weighed amounts of mucosa (1 – 3 g) were thoruoghly grinded with glass beads for 15 min, the cells were then ultrasonicated and completely lysed in 10-15 mL of distilled water, ultracentrifuged at 12000g for 30 min for elimination of the cell membranes and other insoluble materials, and the CA activity in the obtained homogenates was assayed with the esterase method, as described above. Calibration/inhibition curves (in the presence of sulfonamide inhibitors) were also used in some cases for comparing CA activity in treated *vs.* untreated animals.[15,16]

Gastric acid secretion parameters BAO (basal acid output), and MAO (maximal acid output after stimulation with histamine) were determined as described by Lambling et al.,[17] in the gastric juice of Beagle dogs with chronic gastric fistulas,[18] by titration with 0.2 N NaOH to pH 7. [18-20] Two animals were used for each compound studied for its antisecretory properties. The above-mentioned secretion parameters were measured before the initiation of the treatment, one, two and three days after the treatment, which consisted in 2 x 500 mg of inhibitor, twice a day, together with the food (12 hours intervals between the administration of the two doses). CA activity in canine gastric mucosa after 3 days of treatment with some of these CA inhibitors was determined in some animals which were sacrificed at the end of the experiment, by the procedure outlined above.

### Results and Discussion

The metal complexes **4-15** included in this study were prepared as previously reported,[7-10] from the sulfonamide sodium salt and metal salts. Their CA inhibitory properties are shown in Table I.

Modifications of gastric acid secretion parameters in dogs with chronic gastric fistulas after 3 days treatment with 2 x 500 mg (twice a day) of sulfonamides **1-3** or their metal complexes **4-15** are shown in Table II.

CA activity isolated from gastric mucosa of dogs sacrificed after the experiments has also been assayed (Table III).

Data of Table I show that *in vitro*, sulfonamides **1-3** as well as their metal complexes **4-15** act as very potent inhibitors of isozymes CA II and CA IV. Isozyme II (the most abundant in gastric mucosa)[2,5] is

Table I: Metal complexes of heterocyclic sulfonamide **1-3**, of type **4-15** and their CA inhibitory properties against the isozymes involved in gastric acid secretion, hCA II and bCA IV. Abbreviations: aaz = sulfonamide deprotonated species of acetazolamide; mza = sulfonamide deprotonated species of methazolamide; eza = sulfonamide deprotonated species of ethoxzolamide; bza = secondary sulfonamide deprotonated species of benzolamide.

| Compound | No | $K_I$ (nM)* | |
|---|---|---|---|
| | | hCA II | bCA IV |
| Haaz (acetazolamide) | **1a** | 12 | 220 |
| Hmza (methazolamide) | **2** | 14 | 240 |
| Heza (ethoxzolamide) | **3** | 4 | 15 |
| Hbza (benzolamide) | **1b** | 5 | 13 |
| $Zn(aaz)_2$ | **4** | 6 | 125 |
| $Zn(mza)_2$ | **5** | 8 | 110 |
| $Zn(eza)_2$ | **6** | 1 | 4 |
| $Mg(aaz)_2$ | **7** | 8 | 140 |
| $Mg(mza)_2$ | **8** | 4 | 120 |
| $Mg(eza)_2$ | **9** | 2 | 6 |
| $Al(aaz)_3$ | **10** | 3 | 90 |
| $Al(mza)_3$ | **11** | 5 | 95 |
| $Al(eza)_3$ | **12** | 1 | 3 |
| $Al(bza)_3(H_2O)_3$ | **13** | 3 | 8 |
| $Cu(aaz)_2$ | **14** | 1 | 3 |
| $Cu(eza)_2(H_2O)_2$ | **15** | 0.9 | 1.7 |

(h = human; b = bovine isozyme); *Esterase method,[14] with 4-NPA as substrate, Tris buffer pH 7.8, 25 °C.

more sensitive to this class of inhibitors as compared to isozyme IV, as already shown in previous communications of our laboratory.[7-10] Ethoxzolamide and benzolamide were the most potent simple inhibitors, followed by acetazolamide and methazolamide. This trend was also maintained for their metal complexes (in which these sulfonamides act as ligands), with the Cu(II) and Al(III) derivatives slightly more active than the corresponding Zn(II) and Mg(II) complexes.

*In vivo* data of Table II show that $H^+$ ion concentration in gastric juice of dogs with chronic gastric fistula severely drops after 3 days of treatment with 2 x 500 mg CA inhibitors of type **1-13**, or combinations of some of these inhibitors (for instance the Al(III) and Cu(II) derivatives, such as for **(10 + 14)** or **(12 + 15)** for example. The decrease is more marked in the case of the metal complexes as compared to the parent sulfonamide. The latter compounds, such as **1a**, **2** and **3**, generally induced a decrease of 27 – 36 % of the produced titratable gastric acid (first two columns of Table II, expressed as percentuals). In the case of the sulfonamide metal complexes, the corresponding decrease was of 44 – 66 % (the least active was the Mg(II) derivative **8**, with a reduction of 44 %; the largest majority of these complexes induced gastric acid reductions of around 60 – 66 %, being almost doubly as effective as compared to the parent sulfonamides). The Al(III) complexes **10**, **12** and **13** were more efficient as compared to the Zn(II) and Mg(II) derivatives, but a combination of aluminum and copper complexes such as **(10 + 14)** or **(12 + 15)**, (Table II) had an even increased activity. Basal acid output (BAO) was also reduced in the metal complex treated animals as compared to the control- or sulfonamide only treated ones (third and fourth columns of Table II, expressed in percentuals). Thus, sulfonamides **1a-3** produced a 44 – 50 % reduction of BAO, after three days of treatment.

Table II: Gastric acid secretion parameters before and 3 days after treatment with CA inhibitors (2 x 500 mg, twice a day), in dogs with gastric fistula (n = 2 for each inhibitor). BAO represents the basal acid output, whereas MAO the maximal acid output after stimulation with histamine (i.v. injection, 0.05 mg/kg b.w.).

| Treatment | $[H^+]$ (mEq/L)* before treatment | $[H^+]$ (mEq/L)* after treatment | BAO* (mEq/h) before treatment | BAO* (mEq/h) after treatment | MAO* (mEq/h) before treatment | MAO* (mEq/h) after treatment |
|---|---|---|---|---|---|---|
| None | 43.5±1.5 | 44.7±2.3 | 5.2±0.3 | 5.1±0.1 | 20.5±0.3 | 20.0±1.9 |
| **1a** | 48.6±2.7 | 32.2±1.6 | 5.9±0.4 | 3.0±0.3 | 22.6±0.5 | 15.1±0.8 |
| **2** | 49.2±3.5 | 35.8±2.9 | 6.0±1.1 | 3.3±0.4 | 21.7±0.9 | 16.0±1.2 |
| **3** | 47.7±3.1 | 30.4±2.0 | 5.6±0.5 | 3.1±0.2 | 23.4±1.3 | 15.8±2.1 |
| **4** | 54.1±4.0 | 21.5±1.9 | 5.9±0.5 | 2.1±0.2 | 26.8±2.5 | 7.4±2.5 |
| **6** | 50.8±3.0 | 17.2±2.4 | 5.5±0.7 | 1.6±0.1 | 25.0±2.9 | 6.1±1.0 |
| **8** | 46.2±4.5 | 25.7±2.2 | 6.3±0.9 | 3.5±0.5 | 24.8±3.1 | 9.9±1.3 |
| **10** | 45.1±3.8 | 20.0±1.4 | 5.8±0.6 | 1.7±0.3 | 23.9±2.6 | 5.4±2.1 |
| **10 + 14**# | 46.8±3.0 | 17.1±0.8 | 6.0±1.2 | 1.1±0.2 | 19.4±0.9 | 4.6±1.1 |
| **12** | 44.5±2.0 | 18.3±2.6 | 5.7±0.9 | 1.4±0.4 | 26.5±0.4 | 4.9±1.0 |
| **13** | 47.1±2.1 | 17.9±1.4 | 5.9±1.1 | 1.3±0.5 | 26.0±0.7 | 4.7±1.2 |
| **12 + 15**# | 45.2±3.7 | 15.2±0.7 | 5.1±0.59 | 0.9±0.2 | 26.8±1.5 | 4.0±0.5 |

*Mean ± standard deviation (n=3).

### The animal was treated with a combination of 450 mg Al(III) complex + 50 mg Cu(II) complex.

The corresponding reductions for the animals treated with the metal complexes were of 44 –82 % (again with the Mg(II) derivative possessing the lowest activity of 44 %). The majority of the investigated compounds reduced BAO with 70 – 82 % as compared to the same parameter in the control animals. The histamine-stimulated secretion (MAO) were also markedly decreased in the animals treated with these CA inhibitors. (fifth and sixth columns of Table II, expressed in percentuals). Thus, sulfonamides 1-3, generally decreased MAO with 26 – 33 % after three days of treatment. In contrast, sulfonamide metal complexes induced a reduction of 60 – 85 % of this parameter. The Al(III), Zn(II) and (aluminum + copper) complex combinations were again the best MAO inhibitors.

Table III: Total CA activity (CA II + CA IV) from canine gastric mucosa after 3 days of treatment with CA inhibitors of type **1-15**.

| Inhibitor | CA activity (EU/mg)* |
|---|---|
| None | 1.28 ± 0.05 |
| **1a** | 0.54 ± 0.04 |
| **3** | 0.46 ± 0.06 |
| **4** | 0.28 ± 0.07 |
| **6** | 0.20 ± 0.05 |
| **(10 + 14)** | 0.11 ± 0.09 |
| **12** | 0.13 ± 0.08 |
| **(12 + 15)** | 0.10 ± 0.06 |

*Mean ± standard deviation (n=3 different samples of the same animal).

### The animal was treated with a combination of 450 mg Al(III) complex + 50 mg Cu(II) complex.

The inclusion of the copper complexes **14** and **15** in this study (in combination with the corresponding aluminum complexes, in order to avoid severe toxicity problems induced by this essential but highly toxic trace element)[21] is due to the following facts. It has been reported[22,23] that treatment with zinc compounds (zinc is involved in a variety of common, chronic or viral diseases) may disturb the copper homeostasis in the organism, leading to depletion of $Cu^{2+}$ ions in many tissues/organs. [21-23] It is thus of crucial importance to supplement this trace element exogenously, when relatively high amounts of zinc derivatives are used in therapy.[23] A copper complex, such as **14** or **15** added to the antacid treatment based on the zinc and/or aluminum sulfonamide complexes reported here, would alleviate/avoid this problem.

Total CA (CA II + CA IV) activity in gastric mucosa of the animals treated as above proved a strong inhibition of these enzymes in the case in which the sulfonamides/metal complexes have been administered for three days (Table III). Thus, 3 days treatment with sulfonamides **1-3** led to a 57-64 % inhibition of the gastric CAs, whereas the metal complexes (alone of in combination) induced a much stronger inhibition, of 78 – 92 % (Table III, data expressed in percentuals).

All these data prompt us to propose the Zn(II), Mg(II) and Al(III) sulfonamide complexes (eventually in combination with trace amounts of the corresponding copper derivatives) as a valuable new class of antacid derivatives, acting probably by a double mechanism: neutralization of hydrogen ions by a normal acid-base neutralization reaction, coupled with a strong inhibition of carbonic anhydrase isozymes present in the gastric mucosa, followed by reduction of formation of $H^+$ ions due to $CO_2$ hydration. It is not to exclude other mechanisms of action of our compounds, such as interaction with the histamine release processes from mastocytes or basophils, since it has been proved that Zn(II) salts of inorganic or organic acid inhibit these processes.[24] On the other hand, histamine is a powerful CA activator,[25,26] and the X-ray crystallographic structure of the histamine-hCA II adduct has only recently been reported by this group.[27] It is thus possible that the strong gastric acid secretion inhibitory properties observed *in vivo* for the metal complexes of heterocyclic sulfonamides, is due to intricate mechanisms correlating their acid neutralizing capacity with the strong CA inhibitory properties, as well as interaction with the histamine activatory properties upon CA and HCl secretion within the stomach. Since gastric ulcers are presently treated with a variety of very efficient therapies (histamine-$H_2$ blockers, $H^+/K^+$-ATP-ase inhibitors, etc)[1c] we do not propose these compounds as a new anti-ulcer therapy, but as an interesting alternative to the classical and widely used antacids, which generally act as simple acid-base neutralizing compounds. Compounds of the type described here should be used alone or in combination with other acid neutralizing compounds (sodium bicarbonate; MgO; ZnO; AlO(OH), etc). A detailed study regarding such combinations is needed.

**References**

1. a) Preceding part of the series: Supuran, C.T., Scozzafava A. (1999) *Metal Based Drugs*, submitted; b) Supuran, C.T. (1994) "Carbonic anhydrase inhibitors", in "*Carbonic Anhydrase and Modulation of Physiologic and Pathologic Processes in the Organism*", Puscas, I. Ed., Helicon, Timisoara, pp. 29-111; c) Puscas, I., Supuran, C.T. (1996) "Farmacologia clinica da ulcera peptica" in "*Aparelho Digestivo*", Coelho J., Ed., MEDSI, Rio de Janeiro, pp. 1704-1734; d) Scozzafava, A., Menabuoni, L., Mincione, F., Briganti, F., Mincione, G., Supuran C.T. (1999) *J. Med. Chem.* **42**, 2641-2650; e) Scozzafava, A., Briganti, F., Mincione, G., Menabuoni, L., Mincione, F., Supuran C.T. (1999) *J. Med. Chem.***42**, 3690-3700

2. Puscas, I., Supuran, C.T. (1994) "Carbonic anhydrase is an important physiological modulator. The pH theory", in "*Carbonic Anhydrase and Modulation of Physiologic and Pathologic Processes in the Organism*", Puscas, I.,Ed., Helicon, Timisoara, pp. 146-175.

3. a) Ljungstrom, M., Vega, F.V., Mardh, S (1984) *Biochim.Biophys.Acta* **769**, 220-230; b) Read, M.A., Read, D.M., Kapuscinski, M., Shulkes, A. (1992) *Regul. Pept.* **40**, 13-27; c) Fleming, R.E., Parkkila, S., Parkkila, A.K., Rajaniemi, H., Waheed, A., Sly, W.S.(1995) *J.Clin.Invest.* **96**, 2907-2913.

4. a) Kapuscinski, M., Shulkes, A (1995) *J.Gastroenterol. Hepatol.* **10**, 405-412; b) Campbell, V.W., Yamada, T. (1991) *Am. J. Physiol.* **260**, G434-439; c) Campbell, V.W., Yamada, T. (1989) *J.Biol.Chem.*, **264**, 11381-11386.

5. a) Puscas, I. (1984) *Ann.N.Y.Acad.Sci.*, **429**, 587-591; b) Puscas, I. (1994). "Treatment of gastroduodenal ulcers with inhibitors of gastric mucosa carbonic anhydrase". In "*Carbonic Anhydrase and Modulation of Physiologic and Pathologic Processes in the Organism*", I. Puscas, Ed., Helicon, Timisoara, pp. 438-450.

6. Lindskog, S., Wistrand, P.J. (1987) " Inhibition of carbonic anhydrase ", in "*Design of Enzyme Inhibitors as Drugs*", Sandler, M.J., Smith, H.J., Eds., Oxford Univ. Press, pp. 698-723.

7. a) Supuran, C.T., Manole, G., Andruh, M. (1993) *J. Inorg. Biochem.* **49**, 97-103; b) Sumalan, S.L., Casanova, J., Alzuet, G., Borras, J., Castiñeiras, A., Supuran, C.T. (1996) *J. Inorg. Biochem.* **62**, 31-39; c) P. Borja, G. Alzuet, J. Server-Carriò, J. Borras, C.T. Supuran, *Main Group Met. Chem.*, **1998**, *21*, 279-292.

8. a) Supuran, C.T. (1995) *Metal Based Drugs* **2**, 327-330; b) Borras, J., Cristea, T., Supuran, C.T. (1996), *Main Group Met. Chem.* **19**, 339-346; c) ) G. Alzuet, J. Casanova, J. Borras, S. Garcia-Granda, A. Gutiérrez-Rodriguez, C.T. Supuran, *Inorg. Chim. Acta*, **1998**, *273*, 334-338.

9 a) Supuran, C.T., Scozzafava, A. (1997) *J Enzyme Inhib.* **12**, 37-51; b) Mincione, G., Scozzafava, A., Supuran, C.T. (1997) *Metal Based Drugs* **4**, 27-34; c) C.T. Supuran, F. Mincione, A. Scozzafava, F. Briganti, G. Mincione, M.A. Ilies, *Eur. J. Med. Chem,* **1998**, *33*, 247-254; d) C.T. Supuran, A. Scozzafava, A. Jitianu, *Metal Based Drugs,* **1997**, *4*, 307-315; e) C.T. Supuran, A. Scozzafava, F. Mincione, L. Menabuoni, F. Briganti, G. Mincione, M. Jitianu, *Eur. J. Med. Chem,* **1999**, *34*, 585-594.

10 Alzuet, G., Ferrer, S., Borras, J., Supuran, C.T. (1994) *Roum. Chem. Quart. Rev.* **2**, 283-300.

11 Brunton, L.L., "Drugs affecting gastrointestinal function". In *The Pharmacological Basis of Therapeutics*, 8th Edition, A.G. Gilman, T.W. Rall, A.S. Nies, P. Taylor Eds., Pergamon Press, New York, 1990, pp. 897-913.

12. Clare, B.W., Supuran C.T. (1999) *Eur. J. Med. Chem.* **34**, 463-474.

13. Eller, M.G., Schoenwald, R.D., Dixson, J.A., Segarra, T. and Barfknecht, C.F. (1985) *J. Pharm. Sci.* **1985**, *74*, 155-160.

14. Pocker, Y., Stone J.T. (1967) *Biochemistry* **6**, 668-679.

15. Supuran, C.T.; Scozzafava, A.; Ilies, M.A.; Iorga, B.; Cristea, T.; Briganti, F.; Chiraleu, F.; Banciu, M.D. *Eur. J. Med. Chem.* **1998**, *33*, 577-595

16. Scozzafava, A.; Briganti, F.;Ilies, M.A.; Supuran, C.T. *J. Med. Chem.*, **2000**, *43*, in press

17. Lambling, A., Bernier, J.J., Badoz-Lambling, J. (1960) *Arch. Mal. App. Dig. Nutr.* **9**, 1000-1007.

18. Kovacs, T.O., Lloyd, K.C., Walsh, J.H. (1996) *Peptides* **17**, 583-587.

19. Uchiyama, K., Wakatsuki, D., Kakinoki, B., Takeuchi, Y., Araki, T., Morinaka, Y. (1999) *J. Pharm. Pharmacol.* **51**, 457-464.

20. Takemoto, Y., Yuki, H., Nishida, A., Ito, H., Kobayashi-Uchida, A., Takinami, Y., Akuzawa, S., Ohta, M., Satoh, M., Semple, G., Miyata, K. (1998) *Arzneimittelforschung* **48**, 403-407.

21. Prohaska, J.R., Brokate, B. (1999) *J. Nutr.* **129**, 2147-2153.

22. a) Aggett, P.J. (1999) *Eur. J. Med. Res.* **4**, 214-216; b) Camakaris, J., Voskoboinik, I., Mercer, J.F.(1999) *Biochem. Biophys. Res. Commun.* **261**, 225-232.

23. a) Sprietsma, J.E. (1999) *Med. Hypotheses* **53**, 6-16; b) Pena, M..M, Lee, J., Thiele, D.J. (1999) *J. Nutr.* **129**, 1251-1260.

24. Marone, G., Findlay, S.R., Lichtenstein, L.M. (1981) *J.Pharm.Exp.Ther.*, **217**, 292-298.

25. a) Supuran, C.T. (1992) *Rev.Roum.Chim.*, **37**, 411-421; b) Supuran, C.T., Balaban, A.T., Cabildo, P., Claramunt, R.M., Lavandera, J.L., Elguero, J. (1993) *Biol.Pharm.Bull.Japan*, **16**, 1236-1239, c) Supuran, C.T., Balaban, A.T. (1994) *Rev.Roum.Chim.*, **39**, 107-113; d) Barboiu, M., Supuran, C.T., Scozzafava, A., Briganti, F., Luca, C., Popescu, G., Cot, L., Hovnanian, N. (1997) *Liebigs Ann./Recueil* 1853-1859.

26. a) Supuran, C.T., Barboiu, M., Luca, C., Pop, E., Brewster, M.E., Dinculescu, A. (1996) *Eur. J.Med.Chem.*, **31**, 597-606; b) Supuran, C.T., Claramunt, R.M., Lavandera, J.L., Elguero, J. (1996) *Biol.Pharm.Bull.Japan*, *19*, 1417-1422; c) Clare, B.W., Supuran, C.T. (1994) *J.Pharm.Sci.*, **83**, 768-773; d) Coltau, M., Puscas, I., Supuran, C.T. (1994) *Rev.Roum.Chim.*, **39**, 457-462.

27. Briganti, F., Mangani, S., Orioli, P., Scozzafava, A., Vernaglione, G., Supuran, C.T. (1997) *Biochemistry* **36**, 10384-10392.

**Received: January 1, 2000 - Accepted: February 1, 2000 -**
**Received in revised camera-ready format: February 2, 2000**

